# The Use of Optical Coherence Tomography in Dental Diagnostics: A State-of-the-Art Review

**DOI:** 10.1155/2017/7560645

**Published:** 2017-07-16

**Authors:** Monika Machoy, Julia Seeliger, Liliana Szyszka-Sommerfeld, Robert Koprowski, Tomasz Gedrange, Krzysztof Woźniak

**Affiliations:** ^1^Division of Orthodontics, Pomeranian Medical University in Szczecin, Ul. Powstańców Wlkp 72, 70-111 Szczecin, Poland; ^2^Division of Orthodontics, Technical University Dresden, Fetscherstrasse 74, 01307 Dresden, Germany; ^3^Department of Biomedical Computer Systems, Faculty of Computer Science and Materials Science, Institute of Computer Science, University of Silesia, Ul. Będzińska 39, 41-200 Sosnowiec, Poland

## Abstract

Optical coherence tomography provides sections of tissues in a noncontact and noninvasive manner. The device measures the time delay and intensity of the light scattered or reflected from biological tissues, which results in tomographic imaging of their internal structure. This is achieved by scanning tissues at a resolution ranging from 1 to 15 *μ*m. OCT enables real-time in situ imaging of tissues without the need for biopsy, histological procedures, or the use of X-rays, so it can be used in many fields of medicine. Its properties are not only particularly used in ophthalmology, in the diagnosis of all layers of the retina, but also increasingly in cardiology, gastroenterology, pulmonology, oncology, and dermatology. The basic properties of OCT, that is, noninvasiveness and low wattage of the used light, have also been appreciated in analytical technology by conservators, who use it to identify the quality and age of paintings, ceramics, or glass. Recently, the OCT technique of visualization is being tested in different fields of dentistry, which is depicted in the article.

## 1. Introduction

Medical imaging is the basis of effective medical diagnosis and is now the mainstream of a dynamically developing branch of science, which is biomedical engineering. Its development started after an accidental discovery of Wilhelm Conrad Roentgen, a professor of physics, who in 1895 observed little fluorescence during his research on electrical discharges and cathode rays. X-radiation turned out to be a fundamental discovery which is still used in medicine today.

Another milestone was the development of the first computed tomography (CT) device by Godfrey Newbold Hounsfield in 1967. The concept of tomography refers to a method that provides images showing sections of the tested structure. The first CT scanner initiated rapid development of medical imaging techniques. A common feature of different types of CT devices is noninvasive imaging of tissue structures and internal organs, as well as their functional parameters. The desire to minimize invasiveness of methods such as biopsy or exploratory surgery, which are painful and may cause deterioration in the patient's condition, was an impetus for the improvement of computed tomography equipment. As a result, completely new technologies were developed, such as magnetic resonance imaging (MRI), ultrasonography (USG), positron emission tomography (PET), single photon emission computed tomography (SPECT), and the latest and more widely used optical coherence tomography (OCT).

The method of optical coherence tomography using interferometry with partially coherent light was first presented in 1991 at the Institute of Technology of the University of Massachusetts [1]. The first in vivo measurements of the section of the human retina were made two years later in Vienna [2]. The first commercial optical tomography device was produced in 1996 by Zeiss-Humphrey [3].

The article depicts the types of optical tomographs and the schematic construction based on the academic knowledge and enunciates the up-to-date knowledge concluded in the articles accessible in the US National Library of Medicine National Institutes of Health (PubMed), Dentistry & Oral Sciences Source EBSCO, and the http://octnews.org website.

## 2. Types of Optical Coherence Tomography

Optical coherence tomography (OCT) uses a beam of partially coherent light to create tomographic images. Currently, there are two basic types of optical coherence tomography: time domain optical coherence tomography (TdOCT) and Fourier domain optical coherence tomography (FdOCT). The former technique was developed in 1991 by the abovementioned group of researchers from the Massachusetts Institute of Technology in the United States [1] for use in ophthalmic diagnosis. It can produce tomographic images of relatively low quality, resulting from long time of measurement, but it does not allow for three-dimensional imaging of objects [4]. Modern optical tomography with detection in the frequency domain (Fourier domain optical coherence tomography) reduces the capture time by more than a hundred times and creates three-dimensional images of the test object.

Optical coherence tomography enables the study of objects that are partially transparent for light from the near infrared range. In the OCT scanner based on this method, the information about the location of scattering (reflecting) layers along the sample beam is contained in the modulation frequency of the light intensity measured as a function of frequency. The electric signal resulting from detection of spectra of interfering beams is called the signal of spectral bands. Nowadays, two methods of practical realization of this type of detection are used. The first is spectral optical coherence tomography (SOCT). The other method is swept source OCT or optical Fourier domain imaging (OFDI) [4]. The common elements, used in both methods (SOCT and OFDI), are fixed reference mirrors (as opposed to time domain OCT).

This improves mechanical stability of the system. An interference image is obtained by the numerical Fourier transform of registered spectral bands. However, the method of detection of an interference signal is different. In SOCT, the light source generates a broadband light beam. A spectrometer is used to detect signals for individual optical frequencies. In OFDI, an ordinary photodetector is used instead of a spectrometer, because the applied fast tunable laser generates light of a narrow spectral line individually for each wavelength.

The recently introduced SS-OCT uses a short cavity swept laser with a tunable wavelength of operation instead of the diode laser used in spectral-domain OCT [5] The SS-OCT has improved image penetration using a wavelength of 1050 nm and has an axial resolution of 5.3 *μ*m and an axial scan rate of 100,000 scans per second. Prototype models could reach faster scan speed of more than 400,000 scans per second [6, 7]. The 12 × 9 mm scan enables simultaneous imaging of the macula, the peripapillary area, and the optic nerve head and the choroidal thickness. The 12 × 9 mm scan comprises 256 B scans each comprising 512 A scans with a total acquisition time of 1.3 s [8] SS-OCT also provides the capability of a wide field up to 12 × 12 mm images [9]. SS-OCT enables clear simultaneous visualization of the vitreous and the posterior precortical vitreous pockets and the choroid and the sclera [10].

## 3. Operating Principle of Optical Coherence Tomography

OCT is a modular device. It consists of coupled hardware components. It contains the software and five basic modules: a partially coherent light source, an imaging apparatus, a measurement head, a module of data processing, and image generation as well as a computer control system. The light source used in the device determines its axial resolution and penetration depth of the light beam. The OCT imaging apparatus module is the central element of the system. This may be any measuring device capable of measuring the reflected or backscattered light with high sensitivity and resolution. Instruments that enable lossless signal transmission are also indispensable.

Other elements of the described OCT system are the measuring head and the system for bringing the probe beam to the test structure. They take different forms, depending on the field of medicine for which they are intended. Their shape also depends on the structure of the imaging apparatus block. The purpose of this module is to acquire measurement data from the imaging apparatus. Another necessary step is analysis of the obtained values, their processing, and presentation. This is achieved through a variety of techniques in the field of image processing, such as noise reduction algorithms, motion and visualization correction algorithms, segmentation, and image resolution enhancement.

**Figure 1:**
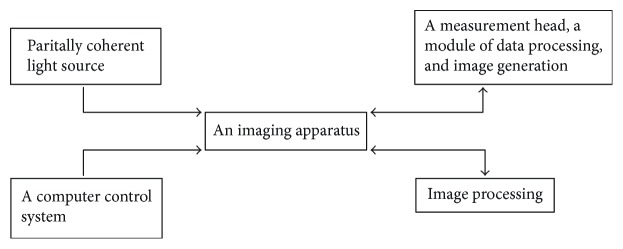
Modular diagram showing the operating principle of OCT.

The computer control system controls the entire OCT scanner. It enables to control scanning the reference arm of the interferometer and synchronize the operation of all components. Moreover, it allows for communication between the apparatus and the image processing block as well as the display of measurement results in real time as it is shown in Figure 1 [11].

OCT imaging is possible by measuring the intensity and time delay of the “echo” of the reflected or backscattered light. The method of OCT imaging is analogous to ultrasonography. However, they differ in terms of data measurement techniques. This is due to the fact that the speed of light is almost one million times greater than the speed of sound and, as a result, the distance measured by OCT is characterized by a much higher time resolution than USG. OCT resolution is 10 *μ*m, and in ultrasonography, it is 150 *μ*m. OCT, on the other hand, has a more limited tissue penetration ability. A light wave in OCT reaches a depth of 2 mm, whereas a sound wave in USG a depth of 10 cm. In the case of USG, electronic detectors can be used for detection of the returning acoustic wave reflected from an object. The use of such devices for detecting light waves is impossible, because the rate of signal changes is too high. The basis of optical tomography is the phenomenon of interference of two partially coherent light beams coming from a single source—the reference beam and the probe beam. Biological objects, such as tissues and organs, are for light waves, the centres with nonuniform distribution of a refractive index. The analysis of interference signal enables to locate the points at which the refractive index changes. These points are situated along the direction of propagation of the probe beam. The graph of reflected wave power density as a function of the position of the reflective point, which is the source of the wave, is called an A-scan. B scans give sagittal scans of the object and C scans—lateral scanning images at a constant depth. Combination of measurement results lying in one plane (numerous parallel directions of the probe beam) creates a two-dimensional image of the section of the test object [12].

**Figure 2:**
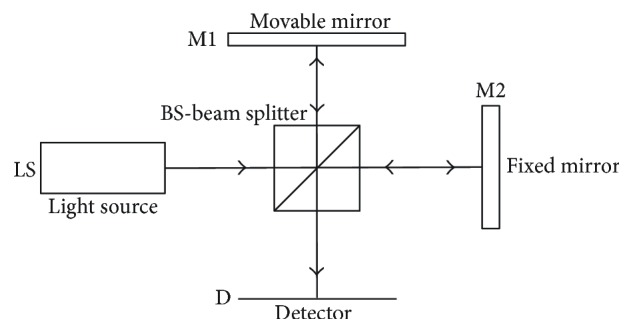
Schematic diagram of the operation of the Michelson interferometer.

The localization of the boundaries of layers with different refractive indices, that is, determination of the waveform of refractive index changes as a function of light beam penetration depth is realized by interferometric distance measurement systems. They use the property of light waves, which is the ability to overlap. This property is dependent on coherence of light. There are two types of light coherence: spatial—defining the phase correlation between wave sequences generated by different points of the light source and time—defining the phase correlation of wave sequences emitted by a single point of the light source at different points in time [13]. The time consistency of light is examined using the Michelson interferometer [14]. The schematic diagram of the operation of the Michelson interferometer is shown in Figure 2.

The light wave incident on the semi-transparent mirror BS (beam splitter) splits into two beams. The light source (LS) changing its direction into perpendicular after passing through BS is reflected by the movable mirror M1, again passes through BS, without changing its direction, and reaches the screen D (detector). The second beam formed by the passage of the primary beam through BS without changing its direction is reflected by the fixed mirror M2, then passes through BS changing the direction into perpendicular, and falls on screen D. The beam incident on the screen forms an interference image.

## 4. The Short History of OCT in Dentistry

Attempts to use optical coherence tomography in dentistry were first made in 1998 by researchers from the Laboratory of Medical Technology of Livermore, California, in collaboration with researchers from the University of Connecticut. In their work, they presented a prototype of dental optical coherence tomography and its in vivo application [15].

The device designed by them scanned hard tissues to a depth of 3 mm and soft tissues to a depth of 1.5 mm, which even now, 14 years after the creation of this sample design, is comparable to the possibilities of the latest generation apparatus. Two years later, the same group of researchers presented the first intraoral scans not only of the hard tissues but also soft tissues of the oral cavity, using another specifically designed CT prototype. In the published work, they demonstrated the possibility of imaging the gum margin, periodontal pockets, and attachments, both epithelial and connective, using an infrared beam of light [16]. The usefulness of optical coherence tomography in the recognition of lesions in the structure of both soft and hard tissues of the oral cavity was also presented in the same year 1998 by experimental and clinical studies conducted by Feldchtein et al. [17], which was actually the first mention of the possibility of OCT examination of hard tissue. In 2000, the same scientific center compared two OCT prototypes having different wavelengths of light: 850 and 1310 nm. Analysis of the quality of scans from individual devices and the evaluation of the possibility of reflecting the anatomical details of the oral cavity showed greater effectiveness of the apparatus using longer wavelengths of light [18]. Five years later, as an experiment, twenty-one dentists were asked to analyze fissure sealants, composite fillings, or tissue enamel based on OCT scans. Despite the lack of knowledge of the techniques of OCT scan interpretation, the dentists who took part in the study obtained clinically acceptable results, which proved the potential clinical application of OCT [19]. The possibility of assessing caries developing under fissure sealants, which is difficult to diagnose, was subject to similar verification. After 90-minute training, doctors assessed the correctness of the enamel structure under 5 different types of sealing materials. When analysing OCT scans, the doctors detected caries more frequently compared with clinical or radiological assessment [20].

In the following years, a leading center dealing with optical tomography became the University of California in San Francisco. A series of articles was published, broadening the knowledge on the aspects of OCT application in conservative dentistry. The described issues were related to imaging of caries incipiens, their remineralization, and monitoring of the progressing or stopped demineralization of the enamel surface or tooth structure underneath fillings [21–29]. The issue of enamel remineralization is still continued [12]. In 2010, an innovative work was presented on attempts of enamel remineralization with chitosan. The penetration depth of chitosan into the enamel structure was evaluated by optical tomography. An attempt of complete enamel remineralization using this method did not prove to be successful, but the exploratory efficiency of the used diagnostic method was once again confirmed [30]. In the same year, the enamel structure of primary teeth was analysed. Since caries is a disease that affects both primary and permanent teeth, the authors verified the effectiveness of the new method of caries diagnosis in the primary dentition. They proved a high potential of optical tomography in paediatric dentistry, as a technique for effective, painless, and noninvasive detection of early tooth decay [31]. The next studies described the effectiveness of optical coherence tomography in monitoring the range and efficiency of infrared and fractional CO2 lasers in caries removal [32–37]. The effectiveness of a diode laser and Nd-YAG laser in the development of root canals during endodontic treatment was also verified [38]. An attempt was also made to use OCT in endodontic in vitro studies [39]. The results of studies evaluating the errors in prosthetic treatment were also published: defects in the structure of the materials used in prosthetic restoration and microleakage at the contact surface of the reconstruction and the tooth as well as the appropriateness of using OCT to control the internal structure of the prosthetic restoration without the need for its removal [34, 40].

Attempts were also made to visualize and measure the length of periodontal ligaments before and during orthodontic tooth movement. Incisors of rats were moved by applying successively varying sizes of forces and then the teeth were removed. The condition of the ligaments was imaged using optical coherence tomography and X-rays. OCT scans showed differences in periodontal ligament arrangement depending on the size of the applied force and their significant twist when using the greatest forces [41]. In subsequent studies, scans of the periodontium were performed and the lengths of both stretched and relaxed ligaments were measured. These structures were imaged using standard radio visual graphic intraoral images. However, they did not prove useful in the evaluation of periodontal elements obstructed in the image by tooth tissues. OCT enabled three-dimensional measurement and multilateral imaging of ligaments. The results obtained when using a CT scanner were different from those obtained by means of standard two-dimensional imaging. Periodontal fibres measured in X-ray images appeared to be much thinner than in reality [42].

Another application of OCT was an attempt to evaluate the salivary pellicle. In order to compare the results and to improve the resolution and specificity of images, an optical coherence microscope (OCM) was used. Salivary pellicle islands were visible in the samples incubated in saliva, which grow into complexes completely covering the enamel surface [43]. The aim of the next study was to evaluate the retention of the biofilm around orthodontic hooks depending on the ligaturing method using OCT and microbiological samples. Both microbiological and optical (OCT) analysis showed a significant difference in biofilm formation depending on the ligaturing method. The hooks ligaturated with elastic elements showed a greater amount of cariogenic *Streptococcus mutans*, whereas metal ligatures showed much less biofilm retention. The study found that optical coherence tomography may also be treated as a full-fledged quantitative indicator of bacterial plaque, which can be quickly and reliably visualized around orthodontic hooks [44]. Similar problems were presented in an ex vivo models. They proved the possibility of calculating the biofilm mass by measuring the distribution of light intensity scattering to a depth of the biofilm. An indirect possibility of characterizing the examined ecosystem on the surface of various types of composite materials was also demonstrated [45]. The study on biofilm imaging, describing the impact of dental calculus, enamel decalcification, and plaque, was an attempt to use optical coherence tomography not only in dentistry but also clinical periodontics. These studies confirmed the possibility of detecting enamel decalcification despite the presence of dental calculus or plaque and their diversification in the scans [46].

Another direction of research using the OCT technique has become the assessment of restorations with composite fillings in conservative dentistry. The study demonstrated, based on analysis of OCT scans, the leakage of composite restorations of enamel defects. The fissures were on average 50 *μ*m. The results were confirmed by X-ray images and optical microscopy. The study resulted in the development of their own spectral CT scanner, which was based on the Michelson interferometer. The created device, as well as the modern optical tomography instrument, divides monochromatic light into two beams, allowing for the reflection of the beams from semi-transparent mirrors and their subsequent interference. Using such a device, the researchers revealed the errors of composite reconstruction in the form of visible pits and fissures at the border between the filling and the cavity wall [47]. Enamel cracks at the border between the enamel and the composite filling reinforced with glass fibre were evaluated in a similar manner [48].The subject of evaluation was also the tightness of three selected composite fillings, cracks of composite reconstruction reinforced with glass fibre, which were imaged using optical coherence tomography (OCT), scanning electron microscopy (SEM), and optical microscopy (OM) [49]. The results enabled to describe the internal cracks of composites, which were not accessible during SEM or OM imaging. It was also observed that the assessment by means of optical coherent tomography required no special sample preparation, making it less expensive compared with the assessment in the scanning electron microscope [50]. In a further step, the efficiency of optical coherence tomography and confocal microscope in the evaluation of composite materials was compared [51].

There are also publications extending the above issue and evaluating marginal adaptation, porosity, and internal integrity of composite fillings. The potential of OCT and high resolution scans, allowing for critical assessment of the structure of fillings, previously inaccessible using common diagnostic methods, has thus been proven [52]. Similar studies evaluating polymerization shrinkage showed significant differences in its size depending on the tested materials [53]. Composite fillings restoring bovine enamel defects and their marginal adaptation with the use of self-etching techniques were also studied. The findings confirmed the thesis that optical coherence tomography is an effective tool in the accurate assessment of tightness of composite fillings [54]. The study of Senawongse et al. [55] made it possible to visualize the adhesive connection between the bonding system and the dentin, analyse carious lesions within the crown and root of the tooth, and assess secondary caries [56, 57]. From a clinical point of view, the studies identifying the relationship between the quality of OCT scans and the level of tooth hydration are very important [58, 59]. It directly affects the strength of the enamel prisms to injuries and the colour of the tissue, which is to be reproduced during conservative or prosthetic restorations. The use of OCT for educational purposes was also presented. The mistakes in the fillings made by dental students were discussed based on performed scans [60].

A further development of work on using an optical scanner and analysis of images was the research which used the potential of OCT to evaluate light scatter and the magnitude of the local refractive index depending on the state of the enamel and dentin. Optical properties of the prisms of the human enamel and dentin tubules were imaged [61].

OCT was also used to evaluate enamel cracks. The results were verified using a stereomicroscope and histological samples of individual enamel layers. Enamel cracks were identified by CT as intensified signals appearing in exactly the same places where damage to the histological samples and stereomicroscopic images was visible. The results showed that OCT very accurately identified cracks and their size, so measurements of the scanned teeth yielded results that were equally reliable to those obtained from stereomicroscopy and histological examination of subsequent enamel layers [62].

In order to improve the quality of OCT scans and facilitate their interpretation, gold nanoparticles were applied. They are normally used as contrast in SEM imaging to visualize the hybrid layer and dentin tubules [63]. This was a significant advancement in dentin imaging because until then only a qualitative and quantitative evaluation of tooth decay had been possible, without distinguishing histological structures [64].

Attempts were also made to use optical coherence tomography in maxillofacial surgery for separating normal and dysplastic fragments of oral epithelium and distinguishing between solid and bullous lesions [65, 66].

The latest studies continue to focus primarily on early diagnosis of caries, assessment of the quality and thickness of dentin, and assessment of dental fillings [67–74]. The precise topics and conclusions of the articles from the last 5 years, according to field of dentistry are summarized in Tables 1, 2, 3, 4, 5, 6, 7, and 8. In the first table, there is set of publications [61, 75–94] that are exposing the facilities of OCT and the possibility of diagnostics in dentistry.

Table 2 collects publications [12, 19, 28, 58, 60, 62, 63, 67, 69–74, 95–148] which show the advancement in cariology and restorative dentistry that has taken place by using the OCT. Publications [39, 56, 149–158] presented in Table 3 are hastening the experiments and the results that were taken in endodontics. The publication [159] contained in Table 4 is the only recent publication connected directly with the pedodontics.  Table 5 present the articles in the field of prosthetics [160–165].  Table 6 collects the articles [45, 65, 66, 166–180] about OCT in periodontology and diagnostics of oral tissues and implantology. The articles about diagnostics in orthodontics are presented in Table 7 [181–188].  Table 8 is collecting the other review articles that can be useful in extending the knowledge about OCT in dentistry.

## 5. Discussion

The common objectives of the discussed studies were increased diagnostic capabilities in the oral cavity, more accurate understanding of physiological and pathophysiological processes related to soft and hard tissues of the oral cavity, and monitoring the effects of treatment.

OCT capabilities commonly applied in many fields of medicine (such as ophthalmology) are not yet fully used in dentistry, mainly due to the low availability of customized intraoral equipment and insufficient range of OCT rays, which penetrate into the tissue to a depth of only a few millimeters depending on the apparatus type. Lesions within the tooth tissue usually reach deeper and are often measured in centimeters, which makes it necessary to perform hundreds or even thousands of scans to illustrate the entire lesion. Latest studies [56, 168, 169] are using the intraoral probes, which show that this obstacle is being slowly eliminated in the intraoral diagnostics.

To maximize the efficiency of the dental diagnostic OCT, the wavelengths of light responsible for generating the image should be subjected to testing. In the near infrared light range, the central wavelength determines the maximum depth of penetration into the tissue due to scattering and absorption properties [71]. A wavelength below 1000 nm provides the greatest imaging efficiency because light scattering properties are similar to the size of tissue particles. Hydrated tissues dissipate much more energy than hard tissues containing a small percentage of water. For this reason, universal dental OCT should offer the possibility of controlling the wavelength depending on the type of the tested tissues. A different wavelength must be used for imaging the periodontal and tooth tissue per se.

However, the technical limitation of the dental OCT is not the only problem. A very important issue is the golden standard that lacks the methodology in many publications. Only few experiments design the study in a manner that compares the obtained results to other more or less conventional methods. There are studies that practice the golden standard by comparing it, for example, to the transverse microradiography [52], microscope [58], standard histopathology [61], confocal laser scanning microscope and light microscopy [70], micro-OCT [74], cone beam computed tomography [82], synchrotron radiation microtomography [103], laser [108], SEM [114], and microfocus X-ray computed tomography [116]. It is important to focus on this topic during analyzing and citing the published results.

Another problem arising in dental diagnosis is the quality of individual teeth. The enamel can vary in its structure in a single subject. Likewise, dental fillings or prosthetic materials having a different composition reflect or absorb light at varying degrees, which has a decisive effect on the image quality and the possibility of its correct interpretation. Materials whose reflectance index is similar to that of the background will give a similar image. In addition to image quality, the possibility of performing objective measurements of the obtained scans is very important. To date, publications have been mainly focused on the possibility of obtaining images of individual structures and their acquisition rate, which is especially important in in vivo studies. The authors of the present paper attempted to develop an algorithm for rapid and accurate measurements of tooth tissues. This algorithm works fully automatically, without any operator intervention, enables to quantify the changes in the structure of enamel, allows for quantitative assessment of the effectiveness of cleaning the tooth surface and the effectiveness of the use of selected methods of enamel development. The analysis time of a sequence of 2D images does not exceed 5 seconds when using the Core i5 CPU M460 @ 2.5 GHz 4 GB RAM. The results of the mean thickness of the tooth enamel and minimum and maximum values as well as standard deviation are analysed automatically and saved to text files ^∗^.txt and Excel ^∗^.xls. Automatic analysis of tooth enamel thickness provides a number of further possibilities. These include area analysis of enamel thickness (for each individual tooth area separately) and enamel texture analysis. Imaging and quantitative measurement of the enamel structure before installation of braces and after their removal enables to expose the tooth tissue damage extent depending on the used brackets and method of attachment. This makes it possible to deduce which brackets and what technique of their installation is the safest for tooth enamel. This solution has been published in work [72]. There are also a few other possibilities for using the quantitative analysis of the intraoral structures and tissue conditions such as dental enamel and dental caries [86], dental abfraction and attrition [98], enamel erosion [101], enamel demineralization [109], thickness of dentin layer [121], and soft tissues [173].

## 6. Conclusions

OCT is a very important tool for the study of various tissues in vivo and in vitro. Despite problems with equipment, the possibility of early diagnosis of caries in conservative dentistry in adults and children has already been proven. It is a unique improvement in relation to X-ray diagnostics exposing patients to X-ray radiation, which is often unable to visualize the early stages of caries.

OCT allows for soft-tissue imaging, which is important in the treatment of periodontal diseases, inaccessible to direct clinical assessment, and offers great perspectives for early diagnosis of lesions in the oral mucosa. Early differentiation of the observed lesion is of great importance in the treatment of a patient due to the frequent occurrence of tumours in the oral cavity. The use of long light waves will also enable the early diagnosis of tumours of the jaw bones.

OCT provides tissue sections in a noncontact and noninvasive manner and allows for real time tissue imaging in situ, without the need for biopsy, histological procedures, or the use of X-rays, so after solving the problems related to the availability and quality of equipment, it will be the method of choice in modern dental diagnostics.

## Figures and Tables

**Table 1:** The OCT facilities and diagnostics in the last 5 years of publications.

Number	Reference number	Author	Title	Significance
(1)	[75]	Shi et al.	Monte Carlo modeling of human tooth optical coherence tomography imaging	This modeling will become a powerful and accurate tool for a preliminary numerical study of the OCT technique on diseases of dental hard tissue in human teeth.

(2)	[76]	Fried et al.	Near-IR imaging of cracks in teeth	Cracks were clearly visible using near-IR imaging at 1300 nm in both in vitro and in vivo images. Cracks and fractures also interfered with light propagation in the tooth aiding in crack identification and assessment of depth and severity.

(3)	[77]	Tom et al.	Near-IR imaging of demineralization under sealants	The wavelength region between 1500–1700 nm yielded the highest contrast of lesions under sealants for near-IR reflectance measurements.

(4)	[78]	Lee et al.	Dental optical coherence tomography: new potential diagnostic system for cracked-tooth syndrome	Crack lines shown in the SS-OCT images had distinct characteristics, and structural crack lines and craze lines could be distinguished in SS-OCT images. Thus, the detection ability of SS-OCT renders it an acceptable diagnostic device for cracked-tooth syndrome.

(5)	[79]	Simon et al.	Near-infrared imaging of secondary caries lesions around composite restorations at wavelengths from 1300–1700 nm	Intensity changes in NIR images at wavelengths ranging from 1300 to 1700 nm correlate with increased mineral loss measured using TMR. NIR reflectance and transillumination at wavelengths coincident with increased water absorption yielded significantly higher (*P* < 0.001) contrast between sound enamel and adjacent demineralized enamel. In addition, NIR reflectance exhibited significantly higher (*P* < 0.01) contrast between sound enamel and adjacent composite restorations than visible reflectance.

(6)	[80]	Chan et al.	Clinical monitoring of smooth surface enamel lesions using CP-OCT during nonsurgical intervention	Even though it appears that most of the lesions manifested little change with fluoride varnish application in the 30 weeks of the study, CP-OCT was able to measure the depth and internal structure of all the lesions including the thickness of the important transparent surface zone located at the surface of the lesions, indicating that CP-OCT is ideally suited for monitoring lesion severity in vivo.

(7)	[81]	Al-Azri et al.	Optical coherence tomography use in the diagnosis of enamel defects	OCT imaging enabled the identification of the type of enamel defect and the determination of the extent of the enamel defects in MIH with the advantage of being a radiation free diagnostic technique.

(8)	[82]	Tezuka et al.	Assessment of cervical demineralization induced by *Streptococcus mutans* using swept-source optical coherence tomography	The gaps along the dentinoenamel junction were additionally observed in SS-OCT. SS-OCT was capable of monitoring the cervical demineralization induced by a cariogenic biofilm and is considered to be a promising modality for the diagnosis of cervical demineralization.

(9)	[83]	Kang et al.	Enhancement of OCT images with vinyl polysiloxane (VPS)	Vinyl polysiloxane (VPS) impression materials which are routinely used in dentistry can be used to enhance the detection of dentinal lesions on tooth occlusal surfaces.

(10)	[84]	Damodaran et al.	Optical coherence tomography-based imaging of dental demineralisation and cavity restoration in 840 nm and 1310 nm wavelength regions	Were comparable with that of the widely used 1310 nm OCT system. In the case of restoration with filler material, the 1310 nm OCT imaging displayed better imaging capacity due to lower scattering than 840 nm imaging.

(11)	[85]	Duma et al.	Handheld scanning probes for optical coherence tomography: developments, applications, and perspectives	Two probes are constructed almost entirely from off-the-shelf components, while a third, final variant is constructed with dedicated components, in an ergonomic design. The handheld probes have unidimensional (1D) galvanometer scanners; therefore, they achieve transversal sections through the biological sample investigated—in contrast to handheld probes equipped with bidimensional (2D) scanners that can also achieve volumetric (3D) reconstructions of the samples. These latter handheld probes are therefore also discussed, as well as the possibility to equip them with galvanometer 2D scanners or with Risley prisms. For galvanometer scanners, the optimal scanning functions studied in a series of previous works are pointed out; these functions offer a higher temporal efficiency/duty cycle of the scanning process, as well as artifact-free OCT images.

(12)	[86]	Mahdian et al.	Tissue characterization using optical coherence tomography and cone beam computed tomography: a comparative pilot study	Within the limitations of this ex vivo pilot study, OCT can reliably differentiate between a range of hard and soft tissues.

(13)	[87]	Bakhsh et al.	Ultrastructural features of dentinoenamel junction revealed by focused gallium ion beam milling	The great potential of cryo-FIB in handling different biological tissues having different physical properties, with great precision and accuracy and minimum artefacts.

(14)	[88]	Oguro et al.	The role of enamel thickness and refractive index on human tooth colours	Enamel affected tooth colour, in which *n* was a statistically significant predictor for tooth colour change.

(15)	[89]	Algarni et al.	Enamel thickness determination by optical coherence tomography: in vitro validation	Human enamel samples were prepared and evaluated with *μ*-CT and PS-OCT and then sectioned and observed via digital transversal light microscopy. For all methods, a standard transversal section (B-scan) in each sample was selected, and the enamel thickness was measured at three predetermined positions using ImageJ analysis software. The results revealed significant high agreement between all tested methods, indicating the potential of PS-OCT as a clinical tool to effectively measure enamel thickness.

(16)	[90]	Wijesinghe et al.	Bio-photonic detection and quantitative evaluation method for the progression of dental caries using optical frequency domain imaging method	The physicians were able to diagnose the tooth volumetric and thickness changes at an initial stage by considering the obtained results as promising threshold parameters, which will be useful to barricade the progression of caries. To enhance the accuracy of the threshold parameters, quantitative (thickness and volumetric) information of multiple in vivo specimens will be evaluated, averaged, and normalized along with clinical trials in future studies.

(17)	[91]	Watanabe et al.	Resolution characteristics of optical coherence tomography for dental use	This study successfully clarified the resolution characteristics of two types of OCTs. The obtained data may be useful for diagnostic purposes, and the glass chart device used in this study may be useful for OCT quality assurance programmes.

(18)	[92]	Kim et al.	Automatic detection of tooth cracks in optical coherence tomography images	The authors were able to distinguish structural cracks, craze lines, and split lines in tooth cracks using SS-OCT images and to automatically detect the position of various cracks in the OCT images. Therefore, the detection capability of SS-OCT images provides a useful diagnostic tool for cracked tooth syndrome.

(19)	[93]	Segarra et al.	Three-dimensional analysis of enamel crack behavior using optical coherence tomography	Crack pattern, tooth type, and the location of the crack on the tooth exhibited a strong correlation. We show that the use of 3D SS-OCT permits for the nondestructive 3D imaging and analysis of enamel crack behavior in whole human teeth in vitro. 3D SS-OCT possesses potential for use in clinical studies for the analysis of enamel crack behavior.

(20)	[94]	Simon et al.	Near-IR and CP-OCT imaging of suspected occlusal caries lesions	Near-IR imaging methods have great potential for improving the early diagnosis of occlusal lesions.

(21)	[61]	Hariri et al.	Effects of structural orientation of enamel and dentine on light attenuation and local refractive index: an optical coherence tomography study.	Unlike enamel, refractive index and OCT signal patterns in dentine vary according to structural orientation, with dentine tubules playing the role. Attenuation of OCT signal intensity was small in enamel. The findings may contribute to a better understanding of the interactions of light with the dental tissue. Precise records of refractive indices and OCT signal patterns may be important for clinical diagnosis of caries and measurement of structural depth for operative purposes using this technology. Effects of dentine structural orientation on refractive index and scattering pattern must be considered when observing human teeth by OCT cross-sectional imaging.

**Table 2:** OCT in cariology and restorative dentistry in the last 5 years of publications.

Number	Reference number	Author	Title	Significance
(1)	[95]	Shimada et al.	Noninvasive cross-sectional imaging of proximal caries using swept-source optical coherence tomography (SS-OCT) in vivo	SS-OCT appears to be a more reliable and accurate method than bitewing radiographs for the detection and estimation of the depth of proximal lesions in the clinical environment.

(2)	[96]	Van Hilsen and Jones	Comparing potential early caries assessment methods for teledentistry	Although MID and CP-OCT were useful in detecting the presence of demineralization, examiners were not able to utilize these devices to adequately assess the depth of the demineralization. This study found that MID and CPOCT did not have markedly superior diagnostic values from simple CAM assessment for use in teledentistry.

(3)	[97]	Nazari et al.	3D assessment of void and gap formation in flowable resin composites using optical coherence tomography	The flowable composite with SDR (stress-decreasing resin) technology performed better than the conventional composite; however, bulk filling a 4 mm-deep cavity will compromise the sealing of the bonding interface regardless of the type of composite. OCT is a unique method of characterizing materials and their behaviors nondestructively and precisely.

(4)	[98]	Mandurah et al.	Monitoring remineralization of enamel subsurface lesions by optical coherence tomography	OCT signal attenuation demonstrated a capability for monitoring changes of enamel lesions during remineralization.

(5)	[99]	de Oliveira Mota et al.	Optical coherence tomography as an auxiliary tool for the screening of radiation-related caries	The OCT technique was able to characterize radiation-related caries, from a morphological point of view. Also demonstrated was its potential benefit for use in the clinical monitoring of radiation-related carious process.

(6)	[100]	Bista et al.	Nondestructive assessment of current one-step self-etch dental adhesives using optical coherence tomography	OCT is a unique tool to nondestructively evaluate the sealing performance of the restoratives through the cavity, provided that cavity walls have a certain minimum inclination with respect to the beam.

(7)	[101]	Park et al.	Assessment of interfacial defects at composite restorations by swept source optical coherence tomography	OCT imaging has the potential to nondestructively assess the interfacial adaptation of composite restorations and to detect internal defects in the layered composite material.

(8)	[102]	Marcauteanu et al.	Quantitative evaluation of dental abfraction and attrition using a swept-source optical coherence tomography system	A valuable tool in the evaluation of the dynamic evolution of ex vivo artificially induced abfractions and attritions is able to measure minute changes in the tooth morphology, having the potential to be employed as an effective tool for monitoring the temporal evolution of dental wear. OCT can offer the possibility of providing in vivo volumetric measurements and identification of fractural lines in dentine. The 2D and 3D pictures prove the OCT ability in the evaluation of dental abfractions and attritions. The system could measure a minimal volume of 2352 *μ*m to 32,352 *μ*m^3^, where each volume is acquired as 25,000 A scans in 2.5 s.

(9)	[103]	Liu and Jones	Evaluating a novel fissure caries model using swept source optical coherence tomography	Despite correctly evaluating the depth, this work showed that the lesion width calculated from SS-OCT reflectivity images did not accurately predict the demineralized width. The relative reflectivity could not accurately determine the mineral density of the demineralized lesions. SS-OCT detected subsurface fissure demineralization and could be used to determine if the decay process was advancing toward the enamel-dentin junction.

(10)	[104]	Chan K. H. et al.	Use of 2D images of depth and integrated reflectivity to represent the severity of demineralization in cross-polarization optical coherence tomography	Calculated lesion depths from OCT were compared with lesion depths measured from histological sections examined using polarized light microscopy. The 2D images of the lesion depth and integrated reflectivity are well suited for visualization of early demineralization.

(11)	[105]	Chew et al.	Measuring initial enamel erosion with quantitative light-induced fluorescence and optical coherence tomography: an in vitro validation study	OCT and QLF were able to detect demineralization after 10 min of erosive challenge and could be used to monitor the progression of demineralization of initial enamel erosion in vitro.

(12)	[106]	Nakajima et al.	Detection of occlusal caries in primary teeth using swept source optical coherence tomography	The results obtained from SS-OCT and conventional visual inspections were compared with those of CLSM. SS-OCT detects both cavitated and noncavitated lesions. The magnitude of sensitivity for SS-OCT was higher than those for visual inspection (sensitivity of visual inspection and SS-OCT, 0.70 versus 0.93 for enamel demineralization, 0.49 versus 0.89 for enamel cavitated caries, and 0.36 versus 0.75 for dentin caries). Occlusal caries of a few clinical cases were observed using SS-OCT in vivo. SS-OCT has a great detecting potential for occlusal caries in primary teeth.

(13)	[107]	Rominu et al.	Zirconia enriched dental adhesive: a solution for OCT contrast enhancement. Demonstrative study by synchrotron radiation microtomography	The present study proved the capability of the OCT method in visualizing the morphology and integrity of zirconia-doped tooth adhesive fillings to be used for a further in vivo tool development.

(14)	[108]	Mandurah et al.	Characterization of transparent dentin in attrited teeth using optical coherence tomography	Physiological changes in transparent dentin that involve deposition of mineral casts in the dentinal tubules lead to lower attenuation of OCT signal. OCT has a potential role to detect transparent dentin on the surface of attrited teeth and can be used in the future as a clinical adjunct tool.

(15)	[109]	Ku et al.	Detection of early changes in caries lesion using QLF-D and OCT	The QLF-D and SS-OCT could detect subtle changes in mineral loss and lesion depth with respect to demineralized time. Furthermore, these devices were useful for monitoring changes in mineral amount and lesion depth.

(16)	[110]	Turkistani et al.	Sealing performance of resin cements before and after thermal cycling: evaluation by optical coherence tomography	OCT could be used for monitoring of composite inlays with several interfacial resin layers. The application of a direct bonding agent in the resin-coating technique improved interfacial sealing and durability of all resin cements.

(17)	[111]	Lee et al.	Automated assessment of the remineralization of artificial enamel lesions with polarization-sensitive optical coherence tomography	PS-OCT can automatically measure the changes in artificial enamel lesion structure and severity upon exposure to remineralization solutions.

(18)	[112]	Chan et al.	A method for monitoring enamel erosion using laser irradiated surfaces and optical coherence tomography	Irradiation of the enamel surface with a pulsed carbon dioxide laser at subablative intensities results in significant inhibition of erosion and demineralization under the acid challenge employed in this study. In addition, these results suggest that it may be feasible to modify regions of the enamel surface using the laser to serve as reference marks to monitor the rate of erosion in vivo.

(19)	[113]	Cara et al.	Evaluation of two quantitative analysis methods of optical coherence tomography for detection of enamel demineralization and comparison with microhardness	Both methods for signal analysis from OCT allowed detection of demineralization with good performance. The AUC-OCT approach enables obtaining a linear relation with the microhardness results, for a quantitative assessment of mineral loss in human teeth.

(20)	[114]	Oancea et al.	Assessment of the sealant/tooth interface using optical coherence tomography	Optical inspection and X-ray investigation revealed no defects, while SS-OCT assesses exactly the position, the nature, and the dimensions of each type of these defects.

(21)	[115]	Damodaran et al.	Development of an electro-optically tuned optical coherence tomography system for imaging dental lesions	The tuning range for LiNbO_3_ and KTP was found to be in the order of few micrometers whereas KTN (potassium tantalate niobate) using the quadratic electro-optic effect is expected to show scanning range of tens of micrometers. KTN based hybrid scanning for dental caries imaging is also planned.

(22)	[116]	Wada et al.	Clinical assessment of non carious cervical lesion using swept-source optical coherence tomography	SS-OCT results confirm that dentin mineral loss and occlusal attrition were associated with larger NCCLs and can be considered as an etiological fact or information and progress of these lesions.

(23)	[117]	Anadioti et al.	Internal fit of pressed and computer-aided design/computer-aided manufacturing ceramic crowns made from digital and conventional impressions	The combination of the digital impression and pressed crown produced the least accurate internal fit.

(24)	[118]	Bortolotto et al.	Failure analysis of adhesive restorations with SEM and OCT: from marginal gaps to restoration loss	When marginal imperfections, or noncontinuous margins, were detected by SEM, also imperfections beneath the surface could be observed at the tooth-restoration interface with OCT. Restoration loss occurred above the borderline of 50% of marginal gaps on enamel and dentin. Marginal discrepancies of adhesive restorations can propagate inside the cavity and lead to restoration loss.

(25)	[119]	Alsayed et al.	Optical coherence tomography for evaluation of enamel and protective coatings	The coatings showed different thicknesses (60–250 micrometers) and various levels of structural and interfacial integrity. OCT could detect a demineralization inhibition zone adjacent to the edge of the fluoride- and calcium-releasing material. Localized demineralization was occasionally observed under thinner coatings. Protection of susceptible enamel surfaces by thin resin-basedbioactive coatings provides protection from demineralization. OCT can be used to nondestructively monitor the integrity of such coatings, as well as enamel changes beneath and adjacent to them.

(26)	[120]	Espigares et al.	Assessment of natural enamel lesions with optical coherence tomography in comparison with microfocus X-ray computed tomography	The images obtained clinically in real time using the dental SS-OCT system are suitable for the assessment of natural subsurface lesions and their surface layer, providing comparable images to a laboratory high resolution *μ*CT without the use of X-ray.

(27)	[121]	Sun et al.	Sensing of tooth microleakage based on dental optical coherence tomography	The results of this study show that microleakage can be detected with oral probing using SS-OCT in vivo. The calculated microleakage length was 401 *μ*m and the width is 148 *μ*m, which is consistent with the related histological biopsy measurements. The diagnosis of microleakage in teeth could be useful for prevention of secondary caries in the clinical treatment plans developed in the field of oral medicine.

(28)	[122]	Park et al.	Assessment of defects at tooth/self-adhering flowable composite interface using swept-source optical coherence tomography (SS-OCT)	Given the high proportion of adhesive defects with the experimental self-adhering flowable composite, its use as the definitive restorative material in class-V cavities must be critically scrutinized and clinical indications must be investigated further with in vitro and in vivo trials.

(29)	[123]	Milly et al.	Surface pre-conditioning with bioactive glass air-abrasion can enhance enamel white spot lesion remineralization	Bioactive glass air-abrasion was used to precondition enamel white spot lesion. Preconditioning increased the average surface roughness of the lesion. An ultrathin, clinically insignificant layer was removed from the lesion surface. Preconditioning enhanced subsequent remineralization using bioactive glass.

(30)	[124]	Min et al.	Evaluation of penetration effect of resin infiltrant using optical coherence tomography	The OCT was the promising quantitative evaluation method for RI penetrated into EC. The OCT would be used as a nondestructive and real-time evaluation method for resin infiltrant penetrated into caries lesion on clinical procedure.

(31)	[125]	Sinescu et al.	Noninvasive quantitative evaluation of the dentin layer during dental procedures using optical coherence tomography	The study demonstrates the usefulness of OCT imaging in guiding such evaluations during dental procedures.

(32)	[126]	Majkut et al.	Validation of optical coherence tomography against micro-computed tomography for evaluation of remaining coronal dentin thickness	We used optical coherence tomography (OCT) and microcomputed tomographic (micro-CT) imaging to scan teeth after deep dentin caries removal. The remaining dentin thickness (RDT) at pulpal horns was measured and compared. A strong correlation was found in measurements between OCT and micro-CT imaging. It was possible to clearly visualize pulp horns with RDT up to 1.5 mm in thickness. A refractive index value of 1.54 is valid to convert optical readings of RDT by OC.

(33)	[127]	Turkistani et al.	Microgaps and demineralization progress around composite restorations	Microgaps forming at the margins of restorations depend on adhesives and significantly contribute to the progress of demineralization around the margins, while fluoride release may decrease the rate of progression.

(34)	[128]	Mota et al.	Optical coherence tomography applied to the evaluation of wear of composite resin for posterior teeth	90% of the restorations of both groups had fractures and/or points of stress concentration, considered niches for early dissemination of new fracture lines.

(35)	[129]	Barbosa et al.	Analysis of photodynamic cream effect in dental caries using optical coherence tomography	The OCT technique demonstrated that cream associated with laser showed the lowest quantitative enamel mineral loses after cariogenic challenge.

(36)	[130]	Makishi et al.	Assessment of current adhesives in class I cavity: nondestructive imaging using optical coherence tomography and microtensile bond strength	Sealing performance was measured in five adhesives by optical coherence tomography. Sealing and bond strength performance within individual specimens were correlated. Interfacial defects increased after thermal aging. Increased interfacial defects tended to decrease the bond strength. Two- and three-dimensional images were useful in assessing bonding performance.

(37)	[74]	Ibusuki et al.	Observation of white spot lesions using swept source optical coherence tomography (SS-OCT): in vitro and in vivo study	SS-OCT appears to be an effective tool for observation of the internal structure of WSLs, enabling quantitative assessment of WSL depth. Such data can be considered in the clinical management of white spot lesion.

(38)	[131]	Yoshimine et al.	Interfacial adaptation of composite restorations before and after light curing: effects of adhesive and filling technique	SS-OCT is a unique method to observe the pre-existing interfacial defects and gaps developed during polymerization, which were found to depend on both placement technique and applied adhesive.

(39)	[132]	Sampaio et al.	Effect of restorative system and thermal cycling on the tooth restoration interface—OCT evaluation	The self-etching adhesive system (CSE) showed better dentin marginal integrity after thermal cycling, compared with the etch-and-rinse (SB2), regardless of the type of resin composite used. Enamel was not affected even after thermal cycling.

(40)	[130]	Makishi et al.	Assessment of current adhesives in class I cavity: nondestructive imaging using optical coherence tomography and microtensile bond strength	Sealing performance was measured in five adhesives by optical coherence tomography. Sealing and bond strength performance within individual specimens were correlated. Interfacial defects increased after thermal aging. Increased interfacial defects tended to decrease the bond strength. Two- and three-dimensional images were useful in assessing bonding performance.

(41)	[133]	Borges et al.	Marginal and internal analysis of preheated dental fissure-sealing materials using optical coherence tomography	Preheated flowable composite provided the best marginal sealing of fissures and internal homogeneity of the material.

(42)	[134]	Dsouza et al.	Assessment of curing behavior of light-activated dental composites using intensity correlation based multiple reference optical coherence tomography	These results show that MR-OCT has the potential to measure the curing time and monitor the curing process as a function of depth. Moreover, MR-OCT as a product has potential to be compact, low-cost, and to fit into a smartphone. Using such a device for monitoring the curing of the resin will be suitable for dentists in stationary and mobile clinical settings.

(43)	[135]	Han et al.	Non-destructive evaluation of an internal adaptation of resin composite restoration with swept-source optical coherence tomography and micro-CT	Micro-CT and SS-OCT may be useful nondestructive methods for evaluating internal adaptation. The microleakage measured by micro-CT was lower than that of SS-OCT; however, the two measurements were relatively high-correlated. When adhesion depends mostly on the dentin surface, a two-step self-etch adhesive system should be considered for long-term longevity.

(44)	[137]	Tom et al.	Near-IR image-guided laser ablation of demineralization on tooth occlusal surfaces	Sequential near-IR reflectance images at 1500–1700 nm can be used to guide a 9.3 *μ*m CO_2_ laser for the selective ablation of early demineralization on tooth occlusal surfaces.

(45)	[138]	Cassimiro-Silva et al.	Mitigation of enamel erosion using commercial toothpastes evaluated with optical coherence tomography	A significant increase in the mean roughness values was observed on eroded surface and also on treated surface as revealed by scanning electron microscopy. The use of SnF_2_/NaF toothpaste was the most effective method for reducing mineral loss. As quantitative methods, OCT and contact profilometry showed no statistical differences. OCT, which was used for this purpose for the first time, has the advantage of being noninvasive and therefore has the potential for clinical application.

(46)	[139]	Dao Luong et al.	Fractography of interface after microtensile bond strength test using swept-source optical coherence tomography	Testing MTBS samples at higher crosshead speeds induced more cracks in dentin. Lining with a flowable composite improved the bonding quality and increased the bond strength. SS-OCT can visualize interfacial cracks after restoration debonding.

(47)	[140]	Ito et al.	Assessment of occlusal fissure depth and sealant penetration using optical coherence tomography	The diagnostic power of SS-OCT was higher than that of visual inspection for fissure depth. Additionally, clear cross-sectional images of sealant penetration into fissures were observed with SS-OCT. SS-OCT can be used to evaluate fissure depth and monitor sealant penetration.

(48)	[141]	Han et al.	Internal adaptation of resin composites at two configurations: influence of polymerization shrinkage and stress	Internal adaptation in a high C-factor cavity was inferior to that in a low C-factor cavity for both conventional and bulk-filled composites. Internal adaptation, polymerization shrinkage, and stress were different among composite materials. Polymerization stress under the compliance-allowed condition showed significant correlations with internal adaptations in high and low C-factor cavities.

(49)	[142]	Horie et al.	Monitoring of cariogenic demineralization at the enamel–composite interface using swept-source optical coherence tomography	The carious demineralization around composite restorations was observed as a bright zone in SS-OCT during the process of bacterial demineralization. SS-OCT appears to be a promising modality for the detection of caries adjacent to an existing restoration.

(50)	[143]	Zhou et al.	Assessment of bacterial demineralization around composite restorations using swept-source optical coherence tomography (SS-OCT)	SS-OCT nondestructively detected demineralization around composite restorations and interfacial gaps created by *S. mutans* biofilm in this in vitro model.

(51)	[144]	de Moraes et al.	Progression of erosive lesions after Nd:YAG laser and fluoride using optical coherence tomography	The OCT technique is promising for diagnosing and monitoring erosive lesion damage; however, further in vitro and in vivo research is needed to improve its use.

(52)	[145]	Ueno et al.	Optical analysis of enamel and dentin caries in relation to mineral density using swept-source optical coherence tomography	Both enamel and dentin demineralization showed significantly higher IS200 and *μμ* than the sound tooth substrate from the sagittal scan. Enamel demineralization showed significantly higher IS200 than sound enamel, even with low levels of demineralization. In demineralized dentin, the *μμ* from the horizontal scan consistently trended downward compared to the sound dentin.

(53)	[146]	Sugita et al.	A pilot study to assess the morphology and progression of non-carious cervical lesions	The dimensional analysis demonstrated notable progression with large variations. The wedge-shaped lesions appeared to show greater *D*_max_ values compared to the saucer-shaped lesions.

(54)	[147]	Schneider et al.	Imaging resin infiltration into non-cavitated carious lesions by optical coherence tomography	Resin infiltration can be increased by optimizing the etching process. Optical coherence tomography provides information about the process and degree of resin infiltration.

(55)	[20]	Holtzman et al.	Ability of optical coherence tomography to detect caries beneath commonly used dental sealants	Dentists were able to detect tooth decay beneath four commonly used dental sealants based on OCT images. Clinical investigations are now underway to determine the usefulness of this approach in vivo.

(56)	[12]	Kang et al.	Nondestructive monitoring of the repair of enamel artificial lesions by an acidic remineralization model using polarization-sensitive optical coherence tomography	This study demonstrated that PS-OCT can be used to nondestructively measure changes in lesion structure and severity upon exposure to an acidic remineralization model. This study also demonstrated that automated algorithms can be used to assess the lesion severity even with the presence of a weakly reflective surface zone.

(57)	[28]	Kang et al.	Nondestructive assessment of early tooth demineralization using cross-polarization optical coherence tomography	Cross-polarization OCT is ideally suited for the nondestructive assessment of early demineralization.

(58)	[58]	Nazari et al.	Effect of hydration on assessment of early enamel lesion using swept-source optical coherence tomography	In summary, the strong relationship found between DH and lesion extent indicates the potential of this method for assessment of early enamel lesion using SS-OCT. However, further studies on DH for evaluation of a wider range of demineralized lesions as well as remineralization, accompanied by a clinically relevant drying method are necessary to optimize the suggested methodology.

(59)	[60]	Shimada et al.	3D evaluation of composite resin restoration at practical training using swept-source optical coherence tomography (SS-OCT)	SS-OCT could detect the internal gaps and voids within the restorations in tomography images synthesized based on the backscatter signal from within the restoration. It is suggested that the SS-OCT is promising diagnostic modality, as well as educational imaging device for the detection of internal gaps in adhesive restorations.

(60)	[62]	Nakajima et al.	Noninvasive cross-sectional imaging of incomplete crown fractures (cracks) using swept-source optical coherence tomography	SS-OCT can clearly discriminate cracks, which appear as highlighted lines due to the scattering of light. The results obtained from the three scanning directions were correlated well with those of the histological sections.

(61)	[63]	Braz et al.	In situ gold nanoparticles formation: contrast agent for dental optical coherence tomography	The results show that the OCT technique, using in situ formed gold nanoparticles as contrast enhancers, can be used to visualize dentin structures in a noninvasive and nondestructive way.

(62)	[67]	Holtzman et al.	Assessment of early occlusal caries pre- and post-sealant application—an imaging approach	This study found that OCT-based imaging combined with a simple diagnostic algorithm accurately assessed the severity of natural early caries on occlusal surfaces in extracted teeth both in the absence and presence of dental sealant. The findings of this study support the clinical use of OCT imaging for assessment and monitoring progression of early noncavitated caries lesions on occlusal surfaces including areas under dental sealants.

(63)	[69]	Sugita et al.	A pilot study to assess the morphology and progression of non-carious cervical lesions	The dimensional analysis demonstrated notable progression with large variations. The wedge-shaped lesions appeared to show greater maximal values compared to the saucer-shaped lesions. With respect to the depth, the wedge-shaped lesions may progress at a greater rate compared to the saucer-shaped lesions.

(64)	[70]	Zhou et al.	Assessment of bacterial demineralization around composite restorations using swept-source optical coherence tomography (SS-OCT)	SS-OCT nondestructively detected demineralization around composite restorations and interfacial gaps created by *S. mutans* biofilm in this in vitro model.

(65)	[71]	Maia et al.	Evaluation of dental enamel caries assessment using quantitative light induced fluorescence and optical coherence tomography	Comparison of the percentage of alteration between optical properties of sound and artificial enamel caries regions showed that OCT processed images through the attenuation of light enhanced the tooth optical alterations more than fluorescence detected by QLF system. QLF versus OCT imaging of enamel caries: a photonics assessment.

(66)	[72]	Horie et al.	Monitoring of cariogenic demineralization at the enamel–composite interface using swept-source optical coherence tomography	The carious demineralization around composite restorations was observed as a bright zone in SS-OCT during the process of bacterial demineralization. SS-OCT appears to be a promising modality for the detection of caries adjacent to an existing restoration.

(67)	[73]	Damodaran et al.	Optical coherence tomography based imaging of dental demineralization and cavity restoration in 840 nm and 1310 nm wavelength regions	Results were comparable with that of the widely used 1310 nm OCT system. In the case of restoration with filler material, the 1310 nm OCT imaging displayed better imaging capacity due to lower scattering than 840 nm imaging.

**Table 3:** OCT in endodontics in the last 5 years of publications.

Number	Reference number	Author	Title	Significance
(1)	[148]	de Oliviera et al.	Detection of apical root cracks using spectral domain and swept-source optical coherence tomography	The detection ability verified for both OCT systems renders them promising tools for the diagnosis of apical microcracks.

(2)	[149]	Brady et al.	A comparison of cone beam computed tomography and periapical radiography for the detection of vertical root fractures in nonendodontically treated teeth	Under the conditions of this ex vivo study, periapical radiographs and CBCT were unreliable for the detection of simulated incomplete VRFs. The widths of the fractures appeared to have an impact on the diagnostic accuracy of CBCT as the detection of VRFs of ≥50 *μ*m was significantly higher than those of <50 *μ*m. The detection of complete fractures was significantly higher for all systems than that of incomplete fractures.

(3)	[150]	Minamino et al.	Nondestructive observation of teeth post core space using optical coherence tomography: a pilot study	In the cementum absent group, the internal structure of the root could be visualized clearly compared with the cementum present group. The root internal structure could be observed by OCT and the image became clearer when cementum was removed.

(4)	[151]	Ding et al.	Application of optical coherence tomography to identify pulp exposure during access cavity preparation using an Er:YAG laser	Swept-source OCT is a useful tool for identifying pulp exposure during access opening with the Er: YAG laser.

(5)	[152]	Chavda R. et al.	Comparing in vivo diagnostic accuracy of digital periapical radiography with cone-beam computed tomography for the detection of vertical root fracture	Both DR and CBCT imaging have significant limitations when detecting vertical root fractures.

(6)	[153]	Iino et al.	Detection of a second mesiobuccal canal in maxillary molars by swept-source optical coherence tomography	SS-OCT imaging is noninvasive, involves no ionizing radiation, and is accurate for the detection of MB2 canals.

(7)	[154]	Majkut et al.	Validation of optical coherence tomography against micro-computed tomography for evaluation of remaining coronal dentin thickness	Further analysis indicated linear regression with a slope of 1.54 and no intercept, closely matching the bulk refractive index of dentin. OCT enables visualization of anatomic structures during deep caries excavation. Exposure of the vital dental pulp because of the removal of very thin remaining coronal dentin can be avoided with this novel noninvasive technique.

(8)	[155]	Minamino et al.	Nondestructive observation of teeth post core-space using optical coherence tomography: comparison with microcomputed tomography and live images	The resulting OCT images were superior for identifying gap formation at the interface, while *μ*CT*μ*CT images were better to grasp the tooth form. Continuous tomographic images from real-time OCT observation allowed successful construction of a video of the resin core build-up procedure.

(9)	[156]	Scotti et al.	Evaluation of composite adaptation to pulpal chamber floor using optical coherence tomography	Composite adaptation to the pulp chamber floor is fundamental for endodontic treatment outcome. Optical coherence tomography is the most noninvasive method to assess interfaces. Less interfacial gaps were observed when flowable resins were used. Any differences between conventional flow and bulk fill composite were shown.

(10)	[157]	Lee et al.	Activity assessment of root caries lesions with thermal and near-IR imaging methods	The PS-OCT algorithm for the automated assessment of remineralization successfully detected the highly mineralized surface layer on both natural and simulated lesions. Thermal imaging provided the most accurate diagnosis of root caries lesion activity. These results demonstrate that thermal imaging and PS-OCT may be ideally suited for the nondestructive root caries lesion activity during a clinical examination.

(11)	[39]	Shemesh et al.	Diagnosis of vertical root fractures with optical coherence tomography	OCT is a promising nondestructive imaging method for the diagnosis of VRFs.

(12)	[56]	Natsume et al.	Estimation of lesion progress in artificial root caries by swept source optical coherence tomography in comparison to transverse microradiography	The OCT showed a potential for quantitative estimation of lesion depth and mineral loss with cavitated dentin lesions in vitro.

**Table 4:** OCT in pedodontics in the last 5 years of publications.

Number	Reference number	Author	Title	Significance
(1)	[159]	Lenton et al.	Clinical cross-polarization optical coherence tomography assessment of subsurface enamel below dental resin composite restorations	CP-OCT imaging may be used to confirm the subsurface marginal integrity below resin composite restorations but with careful consideration of limitations of the imaging modality. CP-OCT imaging may be a useful adjunct to clinical visual investigation to confirm that a composite margin has a sound and well-adapted interface.

**Table 5:** OCT in prosthetics in the last 5 years of publications.

Number	Reference number	Author	Title	Significance
(1)	[158]	Park et al.	Digital technique for in vivo assessment of internal and marginal fit of fixed dental prostheses	Digital approaches to assess the misfit of fixed dental prostheses have been limited to in vitro evaluation. The present article describes a fully digital technique for the in vivo assessment of the fit of fixed dental prostheses by means of a chairside optical scanner and software for three-dimensional (3D) analysis. The 3D digital capture is performed in 3 steps: an extraoral scan of the restoration, an intraoral scan of the abutment tooth, and an intraoral registration scan of the restoration positioned on the abutment tooth.

(2)	[160]	Lin et al.	Examination of ceramic restorative material interfacial debonding using acoustic emission and optical coherence tomography	Sustainable cyclic load stresses in ceramic/dentin-bonded specimens were substantially lower than the measured SBS. Predicted S–N curve showed that the maximum endured load was 4.18 MPa passing 10^6^ fatigue cyclic.

(3)	[161]	Lin et al.	Examination of ceramic/enamel interfacial debonding using acoustic emission and optical coherence tomography	The acoustic emission technique combined with OCT MPa images as a preclinical assessment tool to determine the integrity of cemented load bearing restored ceramic material. Sustainable cyclic load stresses in ceramic/enamel-bonded specimens were substantially lower than the measured SBS. Predicted S–N curve showed that the maximum endured load was 10.98 (about 34.48 N) passing 10^6^ fatigue cyclic.

(4)	[162]	Madaras et al.	Material defects in ceramic crowns identification by optical coherence tomography and microCT	OCT technology can be considered an early diagnosis method of faults contained in the table structure of the ceramic crowns before inserting them in the oral cavity, by reducing the risks of a prosthetic treatment.

(5)	[163]	Fernandes et al.	Optical coherence tomography investigations of ceramic lumineers	The OCT is an effective and promising method to clinical evaluation of the cementing line in lumineers.

(6)	[164]	Gabor et al.	OCT evaluation of single ceramic crowns: comparison between conventional and chair-side CAD/CAM technologies	The marginal accuracy of all ceramic crowns fabricated with digital impression and the CAD/CAM technique is superior to the conventional impression technique.

(7)	[165]	Türk et al.	Comparison of the marginal adaptation of direct and indirect composite inlay restorations with optical coherence tomography	Within the limitations of this in vitro study, marginal discrepancies of inlay restorations were quantitatively and noninvasively evaluated by the OCT system. The following conclusions may be drawn: direct inlays presented smaller marginal gap values than indirect inlays. The marginal gap values were increased for all restorations after cementation.

**Table 6:** OCT in periodontology and diagnostics of oral tissues and implantology in the last 5 years of publications.

Number	Reference number	Author	Title	Significance
(1)	[166]	Gladkova et al.	Evaluation of oral mucosa collagen condition with cross-polarizationoptical coherence tomography	The OCT signal SD in cross-polarized images reflects two boundary conditions of collagen disorganization, namely, loss of fibre properties at active inflammation which attenuates the signal and fibrosis that occurs due to synthesis of a new remodeled collagen which amplifies the OCT signal.

(2)	[167]	Weber et al.	Towards a bimodal proximity sensor for in situ neurovascular bundle detection during dental implant surgery	The proximity to the neurovascular bundle can be tracked in real time in the range of a few millimeters with NIR signals, after which higher resolution imaging OCT to provide finer ranging in the submillimeter distances.

(3)	[168]	Kikuchi et al.	Evaluation of the marginal fit at implant-abutment interface by optical coherence tomography	OCT appeared as an effective tool for evaluating the misfit of implant-abutment under thin layers of soft tissue.

(4)	[169]	Mota et al.	Non-invasive periodontal probing through Fourier-domain optical coherence tomography	Regarding the ability of the two OCT systems to visualize periodontal structures, the system operating at 1325 nm shows a better performance, owing to a longer central wavelength that allows deeper tissue penetration. The results with the system at 930 nm can also be used, but some features could not be observed due to its lower penetration depth in the tissue.

(5)	[170]	Boadi et al.	Imaging of 3D tissue-engineered models of oral cancer using 890 and 1300 nm optical coherence tomography	890 nm OCT retains some of its known advantages of higher contrast between anatomical tissue layers when used to observe dysplastic and malignant 3D oral mucosa constructs. However, 1300 nm OCT is confirmed to possess a greater ability to image the full thickness of the model epithelia, and in particular, it is more suited to imaging through the keratinized layer.

(6)	[171]	Sanda et al.	The effectiveness of optical coherence tomography for evaluating peri-implant tissue: a pilot study	Cement remnants at the submucosal area can be detected in some cases, which can be helpful in preventing peri-implant diseases. Still, though there are some restrictions to its application, OCT could have potential as an effective diagnostic instrument in the field of implant dentistry as well.

(7)	[172]	Damodaran et al.	Non-invasive detection of periodontal loss of attachment using optical coherence tomography	The conventional time domain OCT system acquisition speed is limited by the speed of the mechanical scanning system. In order to overcome this issue, a novel electro-optic-based scanning system is proposed and demonstrated.

(8)	[173]	Fernandes et al.	Monitoring the gingival regeneration after aesthetic surgery with optical coherence tomography	OCT is an efficient method in the evaluation of regeneration gingival.

(9)	[174]	Augustine et al.	Optical coherence tomography in oral cancer detection	OCT can pinpoint epithelial changes; this imaging tool has sought potential broad applications in other mucosal lesions such as vesiculobullous and vascular lesions. The possibility of this application for bone-related disease imaging is an interesting research prospect. Future research should focus on the suitable wavelength of the light source of OCT for better observation of oral diseases. Faster and higher resolution OCT systems may replace the need for biopsies in many situations in the near future.

(10)	[175]	Negrutiu et al.	Assessment of dental plaque by optoelectronic methods	The biofilm network was dramatically destroyed after the professional dental cleaning. OCT noninvasive methods can act as a valuable tool for the 3D characterization of dental biofilms.

(11)	[176]	Fernandes et al.	In vivo assessment of periodontal structures and measurement of gingival sulcus with optical coherence tomography: a pilot study	OCT has the potential to be a reliable tool for in vivo periodontal tissues evaluation and for reproducible sulcus depth measurements in healthy sites. Further technological advances are required to reduce the procedure time and promote evaluation of posterior oral regions.

(12)	[177]	Salehi et al.	Characterization of human oral tissues based on quantitative analysis of optical coherence tomography images	These OCT features can reliably differentiate between a range of hard and soft tissues and could be extremely valuable in assisting dentists for in vivo evaluation of oral tissues and early detection of pathologic changes in the tissues.

(13)	[178]	Englund et al.	Assessing the dynamic biofilm removal of sulfonated phenolics using CP-OCT	This novel CP-OCT flow cell assay has the potential to examine rapid interactions between antibiofilm agents and tooth like surfaces.

(14)	[179]	Bordin et al.	Optical coherence technology detects early signs of peri-implant mucositis in the minipig model	Development of clinical applications of OCT imaging for early diagnosis of mucositis could lead to therapeutic interventions to reduce one of the causes of implant failure.

(15)	[180]	Kim et al.	Improved accuracy in periodontal pocket depth measurement using optical coherence tomography	OCT was able to visualize periodontal pockets and show attachment loss. By calculating the calibration factor to determine the accurate axial resolution, quantitative standards for measuring periodontal pocket depth can be established regardless of the position of periodontal pocket in the OCT image.

(16)	[45]	Chen et al.	Quantifying dental biofilm growth using cross-polarization optical coherence tomography	CP-OCT has the ability to nondestructively monitor biofilm growth and elucidate the growth characteristics of these microcosms on different dental material compositions. CP-OCT was able to quantify the mass of the biofilm by measuring the overall depth-resolved scattering of the biofilm.

(17)	[65]	Adegun et al.	Quantitative analysis of optical coherence tomography and histopathology images of normal and dysplastic oral mucosal tissues	Quantitative differentiation of normal and dysplastic lesions using OCT offers a noninvasive objective approach for localizing the most representative site to biopsy, particularly in oral lesions with similar clinical features.

(18)	[66]	Adegun et al.	Quantitative optical coherence tomography of fluid-filled oral mucosal lesions	The differentiation of normal and fluid-filled areas using individual SID values yielded both a sensitivity and specificity of approximately 80%. OCT complemented by SID analysis provides a potential in vivo clinical tool that would enable noninvasive objective visualization of the oral mucosa.

**Table 7:** OCT in orthodontics in the last 5 years of publications.

Number	Reference number	Author	Title	Significance
(1)	[181]	Koprowski et al.	Automatic method of analysis of OCT images in the assessment of the tooth enamel surface after orthodontic treatment with fixed braces	This paper presents an automatic quantitative method for the assessment of tooth enamel thickness captured on the OCT scans. This method has proven to be an effective diagnostic tool that allows evaluation of the surface and cross section of tooth enamel after orthodontic treatment with fixed thin-arched braces and proper selection of the methodology and course of treatment.

(2)	[182]	Seeliger et al.	Enamel thickness before and after orthodontic treatment analysed in optical coherence tomography	The range of variations in the enamel thickness after treatment with fixed thin-arched braces is not subjected to modification of a factor such as the type of adhesive system. The OCT is an effective diagnostic tool to evaluate the thickness of the enamel tissue before and after the completed orthodontic treatment.

(3)	[183]	Pithon et al.	Effectiveness of fluoride sealant in the prevention of carious lesions around orthodontic brackets: an OCT evaluation	Pro Seal sealant alone or combined with brushing and/or brushing and the use of a mouthwash with fluoride was more effective in protecting enamel, in comparison to brushing alone.

(4)	[184]	Leão Filho et al.	Enamel quality after debonding: evaluation by optical coherence tomography	The results demonstrated that enamel fractures were observed only in the samples bonded with ceramic brackets, and the type of pliers did not influence the incidence and extent of enamel damage. Moreover, the type of debonding technique (with side-cutting pliers or anterior bracket removal pliers) and the type of bracket did not influence the amount of adhesive remaining after debonding. The burs at low speed removed the remaining adhesive more effectively during cleanup procedures.

(5)	[185]	Pithon et al.	Effectiveness of varnish with CPP–ACP in prevention of caries lesions around orthodontic brackets: an OCT evaluation	The major limitation of this study is that it is a study in which demineralization was obtained with the use of chemical products and did not occur due to the presence of *Streptococcus mutans* and its acid byproducts. Application of CPP-ACP-containing varnish irrespective of being associated with brushing and mouthwash, or not, reduced depth of caries lesions around orthodontic brackets.

(6)	[186]	Nee et al.	Longitudinal monitoring of demineralization peripheral to orthodontic brackets using cross polarization optical coherence tomography	CP-OCT was able to measure a significant increase in demineralization (*P* < 0.0001) at the base of orthodontic brackets over a period of 12 months.

(7)	[187]	Isfeld et al.	Assessing near infrared optical properties of ceramic orthodontic brackets using cross-polarization optical coherence tomography	Noninvasive, near infrared (NIR) cross-polarization optical coherence tomography (CP-OCT) has potential to effectively image through portions of ceramic brackets; however, further investigation into the optical effects of resin integration in the base portion of the brackets is warranted.

(8)	[188]	Leão Filho et al.	Optical coherence tomography for debonding evaluation: an in-vitro qualitative study	The analysis of the two-dimensional and three-dimensional images allows observation and evaluation of adhesive remnants, enamel damage, and superficial aspects of enamel from different methods of adhesive remnant removal. The 2D optical coherence tomography analysis allows in-depth observation of the adhesive remnant layer. Optical coherence tomography can be a powerful tool for academic and clinical applications for the evaluation of debonding procedures.

**Table 8:** OCT in dentistry review articles in the last 5 years.

Number	Reference number	Author	Title	Significance
(1)	[189]	Clarkson et al.	Optical technology: an update on optical coherence tomography in dentistry	The technique of optical coherence tomography is considered to be significant since the technology involved allows imaging using light to around 2-3 mm in the teeth and can, for example, allow the extent and progression of carious lesions to be determined.

(2)	[190]	Gupta et al.	Optical coherence tomography: a new era in dentistry	It can be used for noninvasive investigations for both in vivo and in vitro structural imaging within the oral cavity.

(3)	[191]	Canjau et al.	Optical coherence tomography for non-invasive ex vivo investigations in dental medicine—a joint group experience	Complementary studies are possible embracing OCT with more traditional methods, such as confocal microscopy and micro-CT. Combination of principles is expected to evolve due to their limitations when considered separately.

(4)	[192]	Benic et al.	Novel digital imaging techniques to assess the outcome in oral rehabilitation with dental implants: a narrative review	New optical imaging techniques may be considered possible approaches for monitoring peri-implant soft tissue health. MRI and ultrasonography appear promising non-ionizing radiation-imaging modalities for the assessment of soft tissue and bone defect morphologies. Optical scanners and OCT may represent efficient clinical methods for accurate assessment of the misfit between the reconstructions and the implants.

(5)	[193]	Singh M. et al.	Optical coherence tomography—a imaging modality in dentistry beyond X-rays	OCT offers noninvasive, noncontact, in vivo, and real-time subsurface images with high-depth resolution. OCT represents a valuable method for investigation and assessment of the health status of soft oral tissues and of hard dental structures. OCT can be used for evaluation of dental treatments reducing their failure rate and saving time and resources, by eliminating incorrect restorations before their insertion in the oral cavity.

(6)	[194]	Hsieh et al.	Dental optical coherence tomography	Dental OCT demonstrates broad applications in soft and hard tissue imaging and early detection of caries, periodontal disease, and oral cancer. OCT can be used for gingiva, periodontal, and mucosa imaging. OCT may also apply in bone-related disease imaging. OCT and PS-OCT represent powerful ability for early diagnosis of caries. Mineral changes at early demineralization stages can be distinguished by PS-OCT. Subgingival calculus can also be detected by OCT. OCT provides images of dental tissue in situ and real-time and allows early detection of many oral diseases, including caries, periodontal disease, and oral cancer.

(7)	[195]	Shimada et al.	Application of optical coherence tomography (OCT) for diagnosis of caries, cracks, and defects of restorations	Describes the use of OCT for detecting dental caries, tooth fractures, and interfacial aps in intraoral restorations. OCT can be a reliable and accurate method and a safer alternative to X-ray radiography.

(8)	[196]	Benic et al.	Novel digital imaging techniques to assess the outcome in oral rehabilitation with dental implants: a narrative review	Optical scanners and OCT may represent efficient clinical methods for accurate assessment of the misfit between the reconstructions and the implants.

(9)	[197]	Colston et al.	Imaging of the oral cavity using optical coherence tomography	The intensity of backscattered light is measured as a function of depth in the tissue. Low coherence interferometry is used to selectively remove the component of backscattered signal that has undergone multiple scattering events, resulting in very high resolution images (<15 microns). Lateral scanning of the probe beam across the biological tissue is then used to generate a 2D intensity plot, similar to ultrasound images. This imaging method provides information that is currently unobtainable by any other means, making possible such diverse applications as diagnosis of periodontal disease, caries detection, and evaluation of restoration integrity.

(10)	[198]	Se-Wook et al.	Study on application to the field of dentistry using optical coherence tomography (OCT)	This review discusses not only the basic principles of operation, types, advantages, and disadvantages of OCT but also the future applications of OCT technology and their potential in the field of dental diagnosis.
